# Case report: Assessment of linguistic, cognitive, and sensory profile competencies in a child with ASD and epilepsy

**DOI:** 10.3389/fpsyg.2023.1307578

**Published:** 2024-01-12

**Authors:** Alejandro Cano-Villagrasa, Nadia Porcar-Gozalbo, Isabel López-Chicheri García, Miguel López-Zamora

**Affiliations:** ^1^UCAM Universidad Católica de Murcia, Murcia, Spain; ^2^Facultad de Ciencias de la Salud, Universidad Internacional de Valencia (VIU), Valencia, Spain; ^3^Departamento de Psicología Evolutiva y de la Educación, Facultad de Psicología y Logopedia, Universidad de Málaga, Málaga, Spain

**Keywords:** ASD, epilepsy, childhood, language, cognition, sensory profile

## Abstract

**Introduction:**

Autism Spectrum Disorder (ASD) and epilepsy pose significant challenges for early diagnosis during childhood. Current scientific literature does not reflect robust action protocols that allow for a detailed screening of difficulties in this population, especially in areas such as language, cognition, and sensory profile. Additionally, detecting epilepsy before the age of 4 is established as a major current public health challenge in our society.

**Objective:**

The aim was to evaluate a patient exhibiting symptoms compatible with both ASD and epilepsy, determining the linguistic, cognitive, and sensory profile through a clinical assessment protocol. Furthermore, the objective included establishing a diagnosis of ASD.

**Method:**

This single-case study (*N* = 1) presents the evaluation of a 7-year-old patient with suspected ASD, experiencing a decline in linguistic and cognitive competencies following a documented epileptic episode. Evaluation was conducted using instruments such as CELF-5, PROLEC-R, WISC-V, ENFEN, PS-2, ADI-R, and ADOS-2.

**Results:**

Following assessment of language, cognition, sensory aspects, and behaviors associated with ASD, the diagnosis of ASD was confirmed in the patient, along with impairments in expressive and receptive language, executive functioning, and alterations in the sensory profile.

**Conclusion:**

Diagnosing ASD and epilepsy, as well as their evaluation, is a complex process requiring interdisciplinary assessment involving a detailed exploration of all functional competencies in individuals with this comorbidity. Future studies should focus on creating and improving existing protocols to develop optimal and effective evaluation strategies for assessing this population during childhood.

## Introduction

Autism Spectrum Disorder (ASD) has become the primary neurodevelopmental disorder in the pediatric population of our society (Crowell et al., 2019). This disorder is characterized by significant impairments in the pragmatic dimension of language and social interaction across various environments in which the individual develops (American Psychiatric Association, 2013). ASD imposes considerable limitations on the integration and autonomy of individuals, affecting their maturation and overall quality of life (May et al., 2017).

ASD often coexists with a broad spectrum of neurological, psychosocial, or educational pathologies and disorders (Stefanski et al., 2021). One of the major neurological diseases that frequently co-occurs with ASD is epilepsy (Kwon et al., 2022). Epilepsy is a neurological disorder originating in the central nervous system, specifically in the cerebral cortex, where episodes of bioelectrical discharges cause damage to brain structures. In individuals with ASD, these disruptions are exacerbated by the inherent challenges of the condition (Guo et al., 2023). For example, Cano-Villagrasa et al. (2023) describe significant impairments in linguistic and cognitive competencies in individuals with ASD, including difficulties in phonology (problems in speech sound articulation and phoneme discrimination), semantics (limited lexical repertoire in both expression and comprehension), morphosyntax (difficulties in verb conjugation and gender and number agreement in grammatical structures), and pragmatics (disruptions in event narration, social interaction, social cognition, and theory of mind), along with delayed acquisition of reading processes involving alterations in reading speed and accuracy (Keller et al., 2017).

Concerning cognition, individuals with ASD and epilepsy experience difficulties in attentional and mnemonic processes. Performance in tasks involving selective, sustained, and divided attention is significantly lower compared to their peers (Tuchman, 2013). According to Strasser et al. (2018), these clinical profiles exhibit cognitive immaturity, making them unable to sustain attention for extended periods. Additionally, Liu et al. (2022) note that attentional difficulties lead to memory alterations, as the individual’s ability to retain environmental information is directly proportional to their attentional capacity. This means that insufficient attention to surrounding stimuli hinders the creation of a sufficiently strong memory trace, affecting later recall. Finally, Bougeard et al. (2021) state that individuals with ASD and epilepsy also show significant alterations in executive functions related to inhibition, emotional control, and working memory—key functions for the child’s proper functional development, especially in processes like learning and behavioral regulation (Accardo and Malow, 2015).

In addition to linguistic-cognitive impairments, current research indicates that individuals with ASD and epilepsy also experience alterations in sensory profiles (Kashefimehr et al., 2018). Studies such as that by Bjørk et al. (2022) suggest that comorbidity with epilepsy intensifies sensory peculiarities in individuals with ASD, leading to significant alterations in dimensions of registration, sensitivity, and sensory avoidance, forming a dysfunctional sensory profile. This results in difficulties in adequately registering sensory stimuli from the environment (Kadwa et al., 2019), heightened sensitivity to stimuli (van Campen et al., 2015), and a tendency to avoid these stimuli, causing aversion and emotional dysregulation when exposed to them (van Andel et al., 2022).

All these dysfunctions in the linguistic, cognitive, and sensory profiles of children with ASD and epilepsy have a negative impact on their developmental maturation. Therefore, this symptomatic situation is of great interest to national and international healthcare systems, as the prevalence of this comorbidity has exponentially increased in recent decades (Lee et al., 2015). Europe is one of the global regions with lower prevalence of ASD and epilepsy, with the World Health Organization estimating approximately 6 million affected Europeans across 53 countries, with an average prevalence of 8.2 affected individuals per 1,000 inhabitants (Tuchman et al., 2010), and an annual incidence of around 300,000 new cases diagnosed (Sharma et al., 2022). Regarding prevalence, some epidemiological studies highlight that 80% of individuals with ASD and epilepsy are children under the age of 10, and another 20% are adolescents aged between 12 and 15. However, there is a high likelihood of bias in these data due to limitations in epilepsy evaluations in the pediatric population with ASD, as several studies indicate late diagnosis (Francis et al., 2013).

In this way, the comorbidity between ASD and epilepsy becomes a condition with a relatively high prevalence, challenging diagnosis, and significant impairments affecting fundamental competencies such as language, cognition, and sensory profile (El Achkar and Spence, 2015). All of this negatively impacts child developmental maturation, compromising the quality of life for individuals with this comorbidity and increasing the demand for detection by healthcare professionals (Anukirthiga et al., 2019). The early detection of epilepsy in children with ASD is crucial in our country’s neuropediatric units, allowing healthcare professionals to design early interventions aimed at significantly improving the child’s prognosis (Chen et al., 2022). Furthermore, early detection of epilepsy in this population could reduce setbacks in the development of linguistic, cognitive, and sensory profile skills for this group (Pastorino et al., 2021). International evaluation protocols for this comorbidity (Sharma et al., 2022) include measuring alterations in language and communication, intelligence, learning, as well as conducting neuroimaging tests such as electroencephalograms (EEG) to detect underlying epileptic activity (Wadhera, 2021). Interdisciplinary collaboration among neurologists, pediatricians, and ASD specialists is essential to ensure accurate diagnosis and the implementation of an appropriate and personalized treatment plan (Ewen et al., 2019; Wadhera and Mahmud, 2022).

### Objective

This case study presents the evaluation of a patient who sought consultation after visiting several healthcare centers without receiving a definitive diagnosis. Following the initial interview, there is suspicion of a diagnosis of ASD with epilepsy, manifesting linguistic, cognitive, and sensory alterations. This work aims to first describe the evaluation of the patient’s linguistic, cognitive, and sensory competencies; secondly, verify the clinical characteristics established in previous diagnoses, and finally, thirdly, describe the process that ultimately led to the definitive diagnosis of ASD and epilepsy.

### Case history

The patient, MR, 7 years, and 8 months old, is referred to our service by the pediatrician from his community’s reference healthcare center after being assessed by the psycho-pedagogical unit at his educational center, consisting of a psychologist, a pedagogue, and a teacher specializing in hearing and language. Until the date of referral, there was no prior diagnosis, although the educational center suspected a Grade 1 ASD diagnosis, which was never considered due to the child’s satisfactory academic performance until the age of 5. However, at 6 years old, during the 1st year of Compulsory Primary Education, the child experienced a significant regression in linguistic and cognitive competencies, raising concerns among the teaching staff. Consequently, he was referred to the psycho-pedagogical assessment unit at the school. This assessment took place over 5 months due to the child’s attentional difficulties, making it challenging to record language and cognition competencies. The results of this evaluation are reflected in the following (Tables 1 2).

**Table 1:** Results in the linguistic performance from the initial evaluation by the psycho-pedagogical team at the educational center.

CELF-5	PD	PE	Percentile	PC	Interpretation
Sentence comprehension	13	4	<0.1	47	Altered
Linguistic concept	17	6	0.1	54	Not altered
Morphosyntax	20	8	0.2	55	Not altered
Related words	6	2	<0.1	46	Altered
Execution of instructions	21	12	2	66	Not altered
Preparation of sentences	24	10	0.5	60	Not altered
Phrase repetition	34	10	0.5	60	Not altered
Reading Comprehension	3	2	<0.1	39	Altered
Pragmatic Skills Profile	103	3	<0.1	42	Altered

PD, Direct Score; PE, Scalar score; PC, Composite Score.

**Table 2:** Results in cognitive performance from initial evaluation by the educational center’s psychopedagogical team.

WISC-V	Sum of scalar scores	Composite scores	Percentile	Interpretation
Verbal comprehension	23	88	45	Medium-Low
Visuospatial	29	94	55	Medium
Fluid reasoning	18	90	50	Medium
Work memory	21	85	42	Medium-Low
Processing speed	26	86	43	Medium-Low
Total intelligence quotient	39	92	55	Medium

After the relevant evaluation, a diversity care plan was initiated at MR’s school, consisting of four educational support sessions per week: two sessions of pedagogical re-education and two sessions of language re-education. Four months later, the neuropaediatrician from the Child and Adolescent Mental Health Unit (USMIA, in Spanish) at his reference hospital conducted a general exploration of the patient’s physical health.

Firstly, a blood test was performed, and the results did not indicate relevant alterations. Secondly, a sleep-deprived electroencephalogram (EEG) was conducted, revealing that MR had generalized spike-and-wave discharges at three cycles per second, confirming episodes of epilepsy causing absences. The neurology professional highlighted the following information: Upon analyzing the sleep-deprived EEG results, distinctive patterns in brain electrical activity were observed. During normal sleep, a significant increase in delta waves, indicative of deep and restorative sleep, was evident. However, after sleep deprivation, a marked increase in beta waves was noted, suggesting heightened brain activity and a response to wakefulness. Additionally, variability in theta waves was observed, indicating the brain’s adaptation to sleep deprivation. This phenomenon may indicate a compensatory response of the central nervous system to maintain a minimum level of cognitive functioning. Moreover, more frequent spikes and sharp waves were recorded after sleep deprivation, suggesting increased neuronal excitability. These changes could be related to heightened neuronal irritability and may have implications for cognitive function and performance. The parents had not previously reported any absence episodes.

Finally, the M-CHAT instrument (Dereu, 2013) was administered to the family, and 10 min of joint play were conducted to observe potential signs or features of ASD in the patient. The M-CHAT results reflected elevated suspicions of ASD (PD > 3), indicating a possible diagnosis of ASD but without confirmation. Therefore, the professional referred MR to the specialized ASD rehabilitation unit where the present work was conducted for outpatient care and ASD diagnosis.

### Diagnostic evaluation

MR attended the unit for a comprehensive evaluation of all linguistic and cognitive competencies. Additionally, an assessment of his intelligence, executive functioning, and sensory profile was conducted for a complete differential diagnosis.

In the initial interview, the family reported that MR’s birth was full-term without relevant perinatal or postnatal complications, allergies, or physical illnesses of note. Regarding his developmental milestones, MR acquired oral language (first words) at the age of 3. Until then, his communication was through gestures and signs. His comprehension was within the normal range for his chronological age, and his parents did not express concerns about this. He began walking at 12 months, with appropriate postural control as expected. His feeding followed a normal course, without the appearance of food aversions to textures, temperatures, or flavors. Toilet training was achieved at 3 years and 3 months. Lastly, in terms of schooling, MR attends a public school from 9:00 to 17:00. The educational professionals did not report significant abnormalities. They described MR as a calm student who keeps up with the class pace, integrating into the school adequately with no difficulties in acquiring basic concepts related to semantic fields of numbers, letters, or common vocabulary. He participates lightly in class activities without opposition, although he tries to avoid them to not be the center of attention. He does not engage in conflicts with his peers, nor does he self-aggress. However, they mentioned that the patient is perfectionistic and takes time to complete most activities presented in the classroom context.

During the family interview, it was revealed that until the age of 5, MR displayed apparently normal developmental progress. His parents did not notice any language, cognitive, or sensory competency alterations. However, they described MR as a solitary but affectionate child. He enjoys playing with cars and drawing but does not pay attention on a single toy. He systematically stacks toys and becomes upset if the arrangement is changed. During play, he does not seek to share with other children, although he occasionally approaches his parents to show them something interesting about an object.

As for family history, none of his relatives has or had a neurodevelopmental disorder or any other relevant alteration. The parenting practices established by the parents appear suitable. The family is permissive and attempts to engage in dialog with MR when he exhibits unwanted or disruptive behavior. The family environment is also competent, and they have support networks for childcare to attend to the child during the parents’ working hours (Monday to Thursday) in the evenings.

Therefore, the objective of this evaluation was to determine linguistic and cognitive performance, as well as establish alterations in the sensory profile of patient MR. Similarly, an assessment of ASD traits was conducted to confirm the diagnosis. For this purpose, an evaluation protocol was devised, as described in Table 3.

**Table 3:** Evaluation protocol for patient MR.

**Assessment of language skills**
CELF-5. Clinical Evaluation of Language Fundamentals – 5 (CELF-5; Wiig et al., 2018).
The Revised Reading Processes Assessment Battery (PROLEC-R; Cuetos et al., 2007).
**Evaluation of cognitive processes and executive functioning**
Wechsler Intelligence Scale for Children - Fifth Edition (WISC-V; Wechsler, 2010).
ENFEN. Neuropsychological Evaluation of Executive Functions in Children (ENFEN; Portellano et al., 2009).
**Sensory profile evaluation**
Sensory Profile-2 (PS-2; Romero-Ayuso et al., 2016).
**ASD Diagnosis**
The Autism Diagnostic Interview – Revised (ADI-R; Rutter et al., 2006).
Autism Diagnostic Observation Schedule, Second Edition (ADOS-2; Lord et al., 2008).

The choice to use a specific set of tests for the evaluation of children with ASD and epilepsy is based on the need to obtain a comprehensive and detailed understanding of the abilities and challenges that each individual faces. Both conditions, ASD and epilepsy, have a unique complexity that requires a comprehensive evaluation to inform personalized and effective intervention strategies. In the language domain, the use of CELF-5 and PROLEC-R is justified not only for their ability to assess language skills but also for their capacity to reveal potential interferences in reading processes, a crucial aspect for academic development and effective communication in children. The inclusion of WISC-V and ENFEN in the assessment makes sense when considering the need to understand not only the overall intelligence quotient but also specific cognitive processes and executive functions. Given the high prevalence of cognitive comorbidities in ASD and epilepsy, these tools are essential for identifying areas of strength and weakness that guide intervention strategies tailored to each child’s individual skills. The relevance of PS-2 is emphasized when considering the influence of sensory regulation on the daily experiences of the child. Atypical sensory responses can significantly impact the quality of life and participation in everyday activities, and their assessment is crucial to inform therapeutic interventions addressing these specific sensitivities. Regarding the diagnosis of ASD, the inclusion of ADI-R and ADOS-2 is justified due to their recognized validity and reliability in evaluating behaviors and social skills characteristic of the autism spectrum. These tools allow for a structured and observational assessment that facilitates the confirmation or exclusion of the ASD diagnosis.

The next section outlines the methodology employed in the evaluation of patient MR.

## Method

### Instruments

#### CELF-5. Clinical evaluation of language fundamentals – 5

An individual clinical instrument designed to identify, diagnose, and monitor language and communication disorders in children and adolescents aged 5 to 15 years. Its Cronbach’s alpha is 0.91 (Wiig et al., 2018).

#### Revised battery for the evaluation of Reading processes

Proposes appropriate tasks for the evaluation of each reading processing module. Its Cronbach’s alpha is 0.86 (Cuetos et al., 2007).

#### Wechsler intelligence scale for children – fourth edition

A comprehensive clinical instrument administered individually to assess the cognitive capacity/intelligence of children aged 6 years 0 months to 16 years 11 months (6:0–16:11). The WISC-V provides composite and subtest scores representing intellectual functioning in specific cognitive domains, as well as a composite score representing overall intellectual ability. It consists of 16 subtests that can be grouped into two general categories: primary or secondary. This test allows determining the intelligence quotient. Its Cronbach’s alpha is 0.89 (Wechsler, 2010).

#### ENFEN. Neuropsychological evaluation of executive functions in children

Evaluates cognitive performance in activities related to executive functions in children and their level of maturity through four tests (interference resistance, path construction, verbal fluency, and ring construction). The Cronbach’s alpha of this test is 0.87 (Portellano et al., 2009).

#### Sensory Profile-2

Evaluates a child’s sensory processing patterns in the context of daily life, based on the theoretical foundation that differences in children’s sensory processing can either help them integrate more into daily activities or manifest difficulties. The age range to assess is from 3 years old to adolescents (14 years and 11 months). Its Cronbach’s alpha is 0.72 (Dunn, 2014).

#### Autism diagnostic interview – revised

A structured interview conducted with the parent or caregiver of a child and an adult with a mental age of at least 2 years, referred for the evaluation of a possible ASD. The interview is divided into five sections: opening questions, communication questions, social development and play questions, repetitive and restricted behavior questions, and questions about general behavior problems. The Cronbach’s alpha for this test is 0.79 (Rutter et al., 2006).

#### Autism diagnostic observation schedule, second edition

A semi-structured set of observations and a series of activities involving the referred individual and a trained examiner. It can be used to evaluate almost anyone suspected of having ASD, from non-verbal 12-month-olds to verbally fluent adults. The Cronbach’s alpha for this test is 0.84 (Lord et al., 2008).

### Procedure

The assessment of patient MR was conducted in 6 one-hour sessions. These sessions were spaced with this duration interval due to the attentional difficulties presented by the patient and to avoid causing cognitive fatigue and introducing biases during the evaluation. MR’s behavior during the assessment was appropriate, as he cooperated adequately. The evaluation process took place in three phases, detailed in Table 4.

**Table 4:** Patient evaluation phases.

Evaluation phase	Session number	Session objective	Tests administered
1	1	Evaluate language and sensory profile	CELF-5
	2		PROLEC-R
			Sensory Profile-2
2	3	Assess cognitive function	WISC-V
	4		ENFEN
3	5	Assess ASD traits	ADI-R
	6		ADOS-2

**Figure 1:**
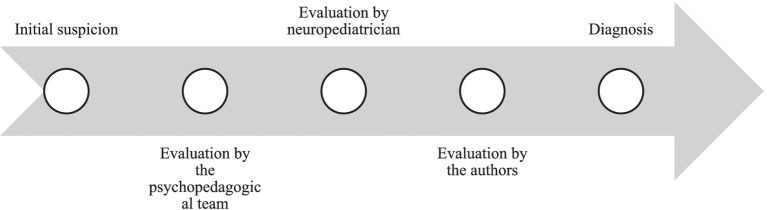
Timeline of patient evaluation procedures.

In addition, in Figure 1, a timeline detailing the evaluation process after the diagnostic suspicion is presented.

## Results

### Language and Reading skills

The results of the CELF-5 test indicate that MR shows significant impairments in various aspects of his expressive and receptive language (Table 5). MR’s language profile is characterized by difficulties in understanding sentences (PE = 5) and oral texts (PE = 5), as well as challenges in establishing word relationships (PE = 3) and pragmatic language skills (PE = 4).

**Table 5:** CELF-5 test results.

	PD	PE	Percentile	PC	Interpretation
Sentence comprehension	18	5	<0.1	49	Altered
Linguistic concept	21	7	0.1	55	Not Altered
Morphosyntax	23	8	0.2	57	Not Altered
Related words	8	3	<0.1	49	Altered
Execution of instructions	24	13	2	68	Not Altered
Preparation of sentences	28	10	0.5	61	Not Altered
Phrase repetition	36	10	0.5	61	Not Altered
Reading Comprehension	6	5	<0.1	49	Altered
Pragmatic Skills Profile	116	4	<0.1	49	Altered

PD, Direct Score; PE, Scalar score; PC, Composite Score.

Similarly, the results of the PROLEC-R instrument reflect difficulties in various reading processes for the patient (Table 6). On one hand, in processes such as identifying equal or different words (PD = 14), reading words (PD = 32), reading pseudowords (PD = 30), respecting punctuation marks during reading (PD = 8), and oral comprehension (PD = 3), patient MR shows slight difficulties compared to his peer group. On the other hand, in processes related to sentence comprehension (PD = 13) and text comprehension (PD = 5), his difficulties are severe.

**Table 6:** PROLEC-R test results.

		Reading performance		
	PD	DD	D	N
Letter name	64			X
Same-Different	14		X	
Lec. Word	32		X	
Lec. Pseudoword	30		X	
Grammar Est.	12			X
Next Score	8		X	
Comp. Prayers	13	X		
Comp. Texts	5	X		
Comp. Oral	3		X	

PD, Direct Score; DD, Severe difficulty; D, Slight Difficulty; N, Normal.

### Cognitive and executive functioning skills

The results of the WISC-V test indicate that MR had an intelligence quotient within the normative range (IQ = 98). However, the ENFEN test reflected that the patient exhibited alterations in executive and cognitive functions (Tables 7 8). MR’s executive functioning scored slightly below the expected level for his age in subtests related to phonological verbal fluency (PD = 4), color trail (PD = 8), and interference (PD = 38). In the rest of the subtests, the patient demonstrates executive functioning within the normotypic range.

**Table 7:** ENFEN test results.

		PD	DC	Interpretation
Verbal fluency	Semantics	14	7	High
	Phonological	4	3	Medium-Low
Trails	Gray trail	17	6	Medium
	Color trail	8	4	Medium-Low
Rings		228	5	Medium
Interference		38	4	Medium-Low

PD, Direct Score; DC, Decatype.

**Table 8:** WISC-V test results.

WISC-V	Sum of scalar scores	Composite scores	Percentile	Interpretation
Verbal comprehension	26	89	49	Medium-Low
Visuospatial	31	96	56	Medium
Fluid reasoning	21	93	53	Medium
Work memory	24	87	45	Medium-Low
Processing speed	28	88	46	Medium-Low
Total intelligence quotient	46	98	64	Medium

### Sensory profile status

The results of the Sensory Profile-2 indicate that MR has scores that fall into the “typical” category. The most significant differences are observed in the behavioral section. Differences are also found in the scores related to avoidance (PD = 30), sensitivity (PD = 60), and registration (PD = 49), with scores placing the patient in a more sensory-sensitive sensory profile than other children of his age (Table 9).

**Table 9:** Sensory Profile-2 test results.

			Interpretation				
	PD	Percentile	Much less than the others	Less than the others	Like the rest	More than the others	Much more than the others
Search	30	10–82			X		
Avoidance	60	>97					X
Sensitivity	50	84–95				X	
Record	49	87–96				X	
Auditory	22	15–80			X		
Visual	13	8–86			X		
Tactile	17	8–86			X		
Motion	11	6–82			X		
Bodily	10	8–89			X		
Oral	22	6–86			X		
Behavioral	33	>96					X
Socio-emotional	38	88–94				X	
Attention	40	>96					X

### Autism Spectrum disorder assessment

Following the evaluation with the ADOS-2 (Table 10) and ADI-R (Table 11) tests, the diagnosis of ASD is confirmed. The results indicate that MR exhibits a set of behaviors consistent with a 299.00 (F84.0) Autism Spectrum Disorder - Level 1, according to the criteria established in the DSM-5 (American Psychiatric Association, 2013), at the time of the assessment.

**Table 10:** ADOS-2 results.

	PD	Interpretation
Social impact	13	Risk
Restricted and repetitive behaviors	6	Risk
Total score	19	ASD Level 1

PD, Direct Score.

**Table 11:** ADI-R results.

	PD	Cut point
Domino A. Qualitative alterations of reciprocal social interaction	27	CPoint = 10 (Risk)
Domain B. Qualitative communication alterations	10	CPoint =8 (Risk)
Domain C. Restricted, repetitive and stereotyped patterns of behavior	5	CPoint =3 (Risk)
Domain D. Developmental alterations evident at 36 months or earlier	2	CPoint =1 (Risk)

PD, Puntuación Directa; CPoint, Cut Point.

### Differential diagnosis

- ASD and Epilepsy vs. Developmental Delay: ASD is characterized by significant deficits in social communication and repetitive behavior patterns. In contrast, developmental delay may manifest more generally, without necessarily including the specific features of ASD, such as a lack of social reciprocity and restricted interests.
- ASD and Epilepsy vs. Language Disorder: In ASD, deficits in social communication are more pronounced, and language disorders may primarily affect language acquisition and use. The presence of specific ASD characteristics, such as a lack of emotional reciprocity and stereotyped language use, should be evaluated.
- ASD and Epilepsy vs. Obsessive-Compulsive Spectrum Disorders (OCSD): Repetitive behaviors in ASD may overlap with obsessive-compulsive behaviors. In ASD, these behaviors are linked to restricted interests, while epilepsy could present compulsive behaviors during or after seizures.
- ASD and Epilepsy vs. Sleep Disorders: Absence seizures may be misinterpreted as sleep-related events. Exploring the child’s sleep history, identifying events during sleep, and evaluating the possible coexistence of sleep disorders that may influence clinical presentation are essential.

### Comorbidity analysis

The comorbidity analysis of patient MR reveals a series of complications that require comprehensive attention. The simultaneous presence of both disorders may increase the risk of mental health issues, such as anxiety and depression. This underscores the need for a thorough mental health assessment to address these concerns and provide appropriate therapeutic interventions. Furthermore, the interaction between ASD and epilepsy can have a significant impact on the child’s cognitive and academic development. The complex relationships between both disorders can influence areas such as attention, learning, and memory. A detailed neuropsychological assessment is essential to understand these areas and design tailored educational strategies. Challenges in communication, inherent to ASD, may be exacerbated by the presence of epilepsy. Epileptic seizures can interfere with effective communication and affect the child’s verbal expression. This highlights the importance of addressing the child’s communicative skills comprehensively. Similarly, challenges in social skills, common in ASD, may be affected by epilepsy. Epileptic seizures can interfere with social interactions and contribute to peers’ perception of strangeness. Specific interventions to improve social skills are essential in this context. Both disorders can also influence the child’s sleep patterns. The complexity of comorbidity may lead to additional difficulties in sleep initiation and quality. Moreover, the simultaneous presence of ASD and epilepsy significantly impacts the family dynamics. Emotional burden, daily challenges, and the need for specialized attention can create family stress. Providing guidance and support to the family is crucial to strengthen resilience and improve the quality of life. Given this complexity, there is a highlighted need for comprehensive intervention addressing both medical and psychosocial aspects. Collaboration among mental health professionals, neurology, special education, and social services is essential to design a personalized treatment plan and provide ongoing support. This coordinated and multidisciplinary approach is key to enhancing the child’s and the family’s quality of life.

## Discussion

Patient MR came to our service with a diagnosis of epilepsy and suspicions of Autism Spectrum Disorder (ASD) without a confirmed diagnosis. The patient did not exhibit significant alterations until the age of 6, when the first recorded epileptic seizure occurred, leading to a decline in linguistic and cognitive abilities, affecting learning and adaptation to daily life contexts. The family reported that during the first 5 years, they did not notice significant difficulties or suspicions of epilepsy or ASD in MR, attributing his isolated and solitary play behaviors to his personality traits. After the epileptic episode and the deterioration of his abilities detected by the educational center, they requested a comprehensive evaluation of linguistic, cognitive, and sensory profile competencies, as well as an evaluation of ASD behaviors that increased in MR.

The assessment had four main objectives. The first was to evaluate language competencies. The second was to assess executive functioning and the possibility of intellectual disability. The third main objective was to determine alterations in the sensory profile. Finally, the fourth objective aimed to confirm the diagnosis of ASD following previous suspicions. The results of the evaluation led to a diagnosis of Grade 1 ASD and epilepsy in patient MR.

This diagnosis was confirmed based on evidence found through the administered instruments. Initially, from the initial family interview and the results of language, reading, executive functioning, and sensory profile tests, it was determined that MR showed linguistic capacity below expectations for his age. Following the epileptic episode, they observed a significant decrease in his vocabulary and comprehension. Additionally, an increase in impulsivity, greater cognitive rigidity, and attention difficulties leading to disruptive behaviors at home were noted. However, they reported that his adaptation to relevant contexts for the child was appropriate, confirming the absence of intellectual disability. Regarding the sensory profile, parents noted that MR tended to avoid certain stimuli and was more sensitive to touch.

After evaluating linguistic, cognitive, and sensory profile competencies related to the ASD diagnosis, MR meets the criteria outlined in the DSM-5 (American Psychiatric Association, 2013). The relevant tests provided evidence of behaviors and conditions sufficient to confirm the diagnosis. The patient exhibited altered linguistic development, with later acquisition of first words (around 3 years old). Furthermore, difficulties in joint play and social relationships were observed. Following this evaluation, contact was made with four specialists in ASD and epilepsy diagnosis, each confirming the diagnosis for MR after reviewing and discussing the assessment results. In summary, behavioral patterns outlined in the DSM-5 (American Psychiatric Association, 2013) for ASD diagnosis in patient MR were confirmed, meeting the following diagnostic criteria: *(A) Persistent deficits in social communication and social interaction across multiple contexts, as manifested by the following, currently or by history, (B) Restricted, repetitive patterns of behavior, interests, or activities, as manifested by at least two of the following, currently or by history, (C) Symptoms must be present in the early developmental period (but may not fully manifest until social demands exceed limited capacities, or may be masked by learned strategies in later life), (D) Symptoms cause clinically significant impairment in social, occupational, or other important areas of current functioning, and (E) These disturbances are not better explained by intellectual disability (intellectual developmental disorder) or global developmental delay.*

In conclusion, cases like MR’s prompt reflect on the difficulty in detecting ASD and epilepsy, establishing an accurate early diagnosis for proper language competency intervention. On one hand, ASD, a complex developmental disorder, manifests differently in each individual, from difficulties in communication and social interaction to repetitive behavior patterns (Lee et al., 2015). These initial manifestations often go unnoticed or are confused with normal developmental phases, leading to delayed and sometimes incorrect diagnoses (Chen et al., 2022). The general lack of awareness about ASD, both among healthcare professionals and society as a whole, exacerbates this situation, delaying early intervention (El Achkar and Spence, 2015).

On the other hand, epilepsy, a neurological condition characterized by recurrent epileptic seizures, manifests in various forms with a wide range of symptoms, complicating its diagnosis (Keller et al., 2017). Episodes may be misinterpreted as neuropsychiatric disorders, delaying the identification of the true cause and, consequently, the application of effective treatments. This diagnostic delay carries significant risks, such as injury or brain damage (Kwon et al., 2022). Therefore, adopting an interdisciplinary approach involving a wide range of health and education professionals is imperative, with speech therapists playing a crucial role in these teams as linguistic impairments are among the most affected competencies in this population. It is essential for healthcare professionals to screen the linguistic, cognitive, and sensory functions of this population to determine their functional status and issue an early diagnosis, initiating multidisciplinary rehabilitation therapy as soon as possible (Cano-Villagrasa et al., 2023).

Investing in scientific research and the development of more precise diagnostic tools also plays a fundamental role in improving the quality of life for individuals diagnosed with ASD and epilepsy. Future research should continue working in this direction to refine an operational evaluation protocol that can detect the regression of some competencies in the population with ASD and epilepsy, such as language and cognition. In this sense, efforts should continue to improve the quality of life and autonomy of the pediatric population with ASD and epilepsy.

## Data availability statement

The raw data supporting the conclusions of this article will be made available by the authors, without undue reservation.

## Ethics statement

The studies involving humans were approved by Universidad Católica San Antonio de Murcia. The studies were conducted in accordance with the local legislation and institutional requirements. Written informed consent for participation in this study was provided by the participants’ legal guardians/next of kin. Written informed consent was obtained from the minor(s)’ legal guardian/next of kin for the publication of any potentially identifiable images or data included in this article. Written informed consent was obtained from the participant/patient(s) for the publication of this case report.

## Author contributions

AC-V: Conceptualization, Data curation, Formal analysis, Funding acquisition, Investigation, Methodology, Project administration, Resources, Software, Supervision, Validation, Writing – original draft, Writing – review & editing. NP-G: Conceptualization, Methodology, Project administration, Writing – original draft, Writing – review & editing. IG: Conceptualization, Data curation, Formal analysis, administration, Resources, Software, Supervision, Validation, Writing – original draft. ML-Z: Conceptualization, Data curation, Methodology, Project administration, Validation, Writing – original draft, Writing – review & editing.
